# Molecular and histopathological evaluation of intestinal mucosal damage associated with giardiasis in naturally infected lambs

**DOI:** 10.5455/javar.2026.m1048

**Published:** 2026-06-22

**Authors:** Zeynep Çelik Kenar, Ayşenur Tural Çifçi, Osman Dağar, Nevin Tuzcu, Hüseyin Karacan, Mehmet Tuzcu

**Affiliations:** 1 Selçuk University, Faculty of Veterinary Medicine, Department of Pathology, Konya, Türkiye; 2 Aksaray University, Faculty of Veterinary Medicine, Department of Pathology, Aksaray, Türkiye; 3 Aksaray University, Eskil Vocational School, Department of Veterinary Medicine, Program of Laboratory and Veterinary Health, Aksaray, Türkiye; 4 Selçuk University, Faculty of Pharmacy, Department of Pharmaceutical Microbiology, Konya, Türkiye; 5 Ankara University, Health Sciences Institute, Ankara, Türkiye

**Keywords:** *Giardia duodenalis*, histopathology, intestine, lamb, real-time PCR

## Abstract

**Objectives:** This study aimed to evaluate the intestinal mucosal alterations in lambs naturally infected with *Giardia duodenalis* at both histopathological and molecular levels, focusing on the relationship between infection severity and gene expression.

**Materials and Methods:** A total of 24 lambs were categorized into four groups (control, mild, moderate, and severe) based on histopathological assessment of intestinal tissue sections. The tissues were examined for villus morphology, epithelial integrity, inflammatory cell infiltration, crypt morphology, and hyperemia. Additionally, the expression levels of *FABP2, TFF3, ALPI, CLDN3*, and *ACTG2* genes were quantified using real-time PCR (qPCR).

**Results:** Histopathological examination revealed progressive villus atrophy, epithelial degeneration, crypt hyperplasia, and lymphoplasmacytic infiltration in the lamina propria, which intensified with infection severity. Molecular analysis showed significant upregulation of *FABP2* and *TFF3* in the mild and moderate groups, suggesting early activation of mucosal repair. In contrast, *ALPI* expression decreased in the moderate and severe groups, indicating impaired epithelial detoxification. *CLDN3* expression was significantly elevated across all infected groups, reflecting a compensatory response to tight junction disruption, while *ACTG2* expression remained unchanged.

**Conclusions:** *Giardia duodenalis* infection in lambs induces complex mucosal pathogenesis. The results demonstrate that the disease involves a simultaneous activation of destructive and regenerative molecular mechanisms, with distinct gene expression patterns correlating with the severity of intestinal damage.

## 1. Introduction

Giardiasis is an important zoonotic disease observed in many mammalian species, particularly in humans and domestic animals [1]. The causative agent, *Giardia duodenalis* (*Giardia lamblia* or *Giardia intestinalis*), localizes in the small intestine, where it causes infection in its trophozoite form and ensures transmission through the cyst form excreted in feces [2]. *Giardia duodenalis* is widespread in both developed and developing countries and may lead to enteric symptoms, malabsorption syndrome, and growth retardation, especially in young individuals [3]. This parasite can be easily transmitted through contaminated drinking water, feed, environmental conditions, and direct contact [2, 3].

Sheep (*Ovis aries*) occupy an important place in the host spectrum of giardiasis and pose a risk to both individual and herd health [4]. Giardiasis cases observed particularly in lambs are characterized by symptoms such as diarrhea, poor weight gain, growth retardation, dull fleece, and decreased appetite [5]. Although the infection is often subclinical, it can lead to significant economic losses due to its negative impact on growth and meat productivity [5, 6].

Molecular studies have revealed that *G. duodenalis* can be divided into several genotypic groups ranging from A to H. Although the non-zoonotic assemblage E is most commonly detected in lambs, the presence of zoonotic assemblages A and B has also been reported. This supports the notion that lambs and sheep may transmit infection to humans through environmental contamination sources [3]. Indeed, recent molecular epidemiological studies have demonstrated that the detection of *Giardia* cysts in dam and stream waters poses a potential risk not only to livestock-handling communities but also to the broader public [3, 7, 8].

Fatty acid-binding protein 2 (FABP2) is a cytoplasmic carrier protein with a molecular weight of 14–15 kDa, abundantly found in intestinal epithelial cells [9]. This protein plays a key role in the intracellular transport of long-chain fatty acids within enterocytes and serves as an important biomarker for the integrity of the small intestinal mucosa [10]. Clinically, beyond its physiological role, the most significant feature of FABP2 is its ability to rapidly enter the bloodstream and other body fluids upon damage to intestinal epithelial cells, allowing its measurement. In this regard, FABP2 is considered a sensitive biomarker for assessing increased intestinal permeability, epithelial injury, and inflammation [11].

Giardiasis is characterized by the attachment of *G. duodenalis* trophozoites to the intestinal mucosa, leading to disruption of villus architecture, enterocyte dysfunction, and cell death. During this process, the integrity of the intestinal barrier is compromised. As a key indicator of intestinal epithelial dynamics, FABP2 reflects the balance between cellular injury and the subsequent mucosal response. Therefore, evaluating the expression levels of FABP2 provides critical data for assessing the severity of protozoal-induced enteropathy and the early stages of epithelial turnover in the small intestine. However, no studies have been found examining intestinal tissue in lambs by qPCR or evaluating the fluctuations in FABP2 expression during giardiasis.

Trefoil Factor Family 3 (TFF3) is a small peptide glycoprotein secreted by goblet cells in the intestinal mucosa [12]. As a member of the trefoil factor family (TFF1, TFF2, TFF3), TFF3 plays an important role in mucus production, epithelial integrity, and mucosal healing. TFF3 is particularly expressed at high levels in the distal ileum and colon [13]. It has been reported that TFF3 expression rapidly increases following mucosal injury, supporting mucosal healing through mechanisms such as apoptosis inhibition and epithelial cell migration [14].

Alkaline phosphatase (ALP) is an enzyme family found in various tissues, among which the intestinal isoform (ALPI) is specifically secreted by enterocytes into the intestinal lumen and plays an important role in maintaining the integrity of the intestinal mucosa [15]. ALPI performs several functions, including detoxification of lipopolysaccharides (LPS) from gram-negative bacteria, limitation of inflammation, preservation of intestinal microbiota balance, and support of epithelial cell regeneration [15, 16]. It has been reported that mucosal or epithelial damage and inflammation in the intestinal epithelium lead to a reduction in ALPI production [16]. Therefore, in *Giardia* infections, the attachment of trophozoites to the epithelium and the resulting damage to enterocytes, as well as disruption of the microvillus structure, may lead to a decrease in ALPI levels.

Claudin-3 (CLDN3) is one of the integral membrane proteins located in the tight junction structures between intestinal epithelial cells [17, 18]. The claudin family is responsible for maintaining mucosal barrier function by regulating paracellular permeability between epithelial cells [17]. CLDN3 is abundantly expressed, particularly in the epithelial cells of the colon and ileum, where it contributes to the tightness of the epithelial barrier [18, 19]. When intestinal barrier integrity is disrupted or an inflammatory environment develops, a decrease in CLDN3 expression and an increase in epithelial permeability occur [20]. As a result, the translocation of pathogens, antigens, and toxins into the systemic circulation becomes facilitated [18, 20]. In *Giardia* infections, trophozoites flatten the microvilli, which also affects the tight junctions between enterocytes [21]. Consequently, it is thought that CLDN3 is impacted, and through feedback mechanisms, interepithelial connections become further compromised [18].

ACTG2 is the gene encoding an actin isoform responsible for the contraction of smooth muscle cells located in the tunica muscularis layer of the gastrointestinal system, playing a crucial role in regulating intestinal motility and peristaltic activity [22]. Under physiological conditions, ACTG2 functions in coordination with the enteric nervous system to maintain the mechanical functions of the intestine [23]. It is known that in chronic giardiasis, not only the intestinal epithelial cells and their intercellular junctions but also the subepithelial tissues are affected. However, this condition primarily results not from the *Giardia* infection itself but from secondary infections that develop due to epithelial damage [24, 25, 26]. In such cases, alterations—either an increase or decrease—in ACTG2 expression during chronic *Giardia* infections may lead to intestinal motility disorders and abnormal changes in contraction rhythm.

The clinical application of intestine-related biomarkers has recently gained traction in veterinary medicine for the objective assessment of mucosal injury. For instance, FABP2 has been identified as a sensitive indicator for detecting intestinal epithelial damage in neonatal calves with diarrhea [27]. Furthermore, similar biomarker profiles have been evaluated in calves with coccidiosis [28] and neonatal lambs with various enteric challenges [29], demonstrating their potential for monitoring intestinal health in ruminants. However, a specific evaluation integrating both the histopathological scoring and molecular gene expression of these biomarkers in natural *Giardia* infections remains a significant gap in the literature. The objective of our study was to evaluate the intestinal mucosal alterations and the expression levels of *FABP2, TFF3, ALPI, CLDN3*, and *ACTG2* genes in lambs naturally infected with *G. duodenalis* at both histopathological and molecular levels.

## 2. Materials and Methods

### 2.1. Ethical approval

The study was reviewed and approved by the Selçuk University Faculty of Veterinary Medicine Experimental Animal Production and Research Center Ethics Committee (SÜVDAMEK) during the meeting held on June 4, 2025, with the decision number 2025/95, under the research project titled “Investigation of Giardia Infection in Lambs in the Konya Region by Histopathological and Molecular Methods”.

### 2.2. Study design

The material of this study consisted of paraffin blocks prepared from small intestine tissues obtained from lambs submitted to the Department of Pathology, Faculty of Veterinary Medicine, Selçuk University, between 2021 and 2024. A total of 24 lambs (*n* = 6 per group) were included in this study. The animals were aged between 2 and 4 months and were predominantly of the Akkaraman and Merino breeds, which are common in the region. The infected groups (mild, moderate, and severe) consisted of naturally infected clinical cases submitted to the Department of Pathology. The control group (*n* = 6) was composed of age and breed-matched healthy lambs obtained from a local private farm, confirmed to be parasite-free through repeated fecal examinations. To ensure the specificity of the molecular and histopathological data, a strict inclusion policy was applied: only lambs that were positive for *G. duodenalis* and negative for other common enteric pathogens, including *Eimeria* and *Cryptosporidium*, were included. Any samples identified with mixed infections were excluded from the study to prevent confounding effects on gene expression profiles and mucosal morphology. According to the clinical history provided by the owners, the lambs included in this study had no reported history of recent treatments. While the nature of naturally infected field cases involves certain inherent variables, inclusion was based on the absence of visible therapeutic intervention at the time of pathological submission.

### 2.3. Histopathological examination

Paraffin blocks were cut into 4–5 µm thick sections using a microtome (Leica RM2125RT) and stained with hematoxylin-eosin (HE) [30]. Following staining, the coverslip-mounted preparations were examined under a binocular light microscope (OLYMPUS, BX51) and photographed with a camera (OLYMPUS, EP50).

### 2.4. Histopathological evaluation

Histopathological evaluation included the evaluation of intestinal villi, epithelial layer, infiltration of the intestinal tract, crypt changes, and hyperemia. The semi-quantitative scoring system for intestinal injury was established by adapting the histomorphological criteria described by Day et al. [31] and Erben et al. [32]. Briefly, villus atrophy, epithelial degeneration, and inflammatory cell infiltration in the lamina propria were scored (0–3) to categorize the severity of infection. This adapted scoring model was specifically tailored to evaluate the typical mucosal alterations associated with protozoal enteritis in ruminants. Evaluations were performed as described in Table 1.

**Table 1.** Scores and definitions used in the histopathological evaluation of the intestines.

Parameter (each scored 0–3)	0 (Control)	1 (Mild)	2 (Moderate)	3 (Severe)
Villi	Normal height/shape	Slight shortening or blunting (≤ 25%)	Marked shortening/tendency to fusion (25–50%)	Severe shortening/fusion/atrophy (> 50%), villi effacement
Epithelial layer	Normal integrity	Superficial desquamation; microvillus loss	Extensive desquamation/erosion foci; enterocyte vacuolization	Widespread erosion/necrosis; extensive epithelial loss
Infiltration in lamina propria	Physiological cell density	Mild, focal lymphoplasmacytic increase	Moderate, diffuse lymphoplasmacytic and/or macrophagic infiltration	Dense, diffuse; neutrophil infiltration, prominent around crypts
Crypt alterations	Normal depth/mitosis	Mild hyperplasia; occasional mitoses	Marked hyperplasia; cryptitis foci	Severe hyperplasia; prominent cryptitis
Hyperemia	Absent	Minimal	Moderate	Severe

### 2.5. Bacteriological examination

During the necropsy of the lambs, bacterial cultures were performed from lymph nodes, liver, and heart tissues on blood agar, nutrient agar, and MacConkey agar. The intestinal paraffin blocks of lambs in which no bacterial growth was observed under aerobic, anaerobic, and microaerophilic incubation conditions were included in the study.

### 2.6. Parasitological examination

In the parasitological examination of the intestinal contents collected during necropsy, samples found to contain *Cryptosporidium* or *Eimeria* oocysts were excluded from the study. Samples that tested positive for *Giardia* were included in the analysis.

### 2.7. Real-time PCR analysis

#### 2.7.1. Total RNA isolation

From each paraffin block, five sections with a thickness of 10 µm were obtained using a separate microtome blade for each block. RNA isolation from the collected tissue sections was performed using the Roche High Pure FFPET RNA Isolation Kit, following the manufacturer’s instructions. The total RNA samples obtained were stored at –70°C.

#### 2.7.2. Obtaining cDNA

cDNA was synthesized from the RNA isolated from tissue samples using the OneScript Plus Synthesis Kit (abm, catalog number: G236) according to the manufacturer’s recommendations.

#### 2.7.3. Real-time PCR

Real-time PCR was performed using a Roche LightCycler^®^ 96 Real-Time PCR System. For this purpose, ABM BlasTaq^™^ 2X PCR MasterMix (ABM, Canada, catalog number: G891) was used. For each target gene, the reaction mixture was prepared using SYBR Green Master Mix as described in Table 4. The primer sequences used in the study are listed in Table 2. The primer sequences for the target genes were designed based on reference sequences registered in the GenBank database. Accordingly, the mRNA sequences of the genes *TFF3* (NM_001114509.2), *ALPI* (NM_173987.2), *CLDN3* (NM_205801.2), *FABP2* (NM_001025332.1), and *ACTG2* (NM_001013592.1) were obtained from NCBI GenBank. Primer design was performed using IDT PrimerQuest and OligoAnalyzer programs. During the design process, parameters such as GC content (40–60%), melting temperature (Tm), amplicon length, and the formation of secondary structures (dimers, hairpins) were taken into account. Primer specificity was verified *in silico* using the NCBI Primer-BLAST tool. The β-actin gene was used as a reference (housekeeping) gene for gene expression analyses. Primer specificity was verified in silico using the NCBI Primer-BLAST tool. Additionally, the specificity of the amplification for each gene was experimentally confirmed by melting curve analysis at the end of each qPCR run, which showed a single specific peak for all target and reference genes.

Each gene was prepared with a separate reaction mixture. For each sample, 15 µl of reaction mixture and 5 µl of cDNA sample were added into the capillaries. The reaction conditions were set as presented in Table 5.

The analysis of gene expression levels was performed using the ^ΔΔ^Ct (Delta Delta Ct) method. For each gene, the expression level was calculated by normalizing the Ct value obtained for the target gene to the Ct value of β-actin obtained from the same tissue [33].

**Table 2.** Primer sequences used in this study*.

β-actin	F: 5’-GGCCACAATGGCTGACCATTC-3’	
	R: 5’-AAGGTGACAGCATTGCTTC-3’	[37]
*FABP2*	F: 5’-GTGTGGAAGCCAAGAGGATT-3’	
	R: 5’-CCAGATGAGGAAAGGAAAGCA-3’	
*TFF3*	F: 5’-GCTCATCCTTCTGCTTCTCTC-3’	
	R: 5’-CTGAGCACGGGAGCTTTATT-3’	
*ALPI*	F: 5’-CAGACCACTCTCATGTCTTCTC-3’	
	R: 5’-GTGTAGGACTTGCTGTCTAAGG-3’	
*CLDN3*	F: 5’-CATCGTCATCGCCATCCTAC-3’	
	R: 5’-ATGGTGATCTTGGCCTTGG-3’	
*ACTG2*	F: 5’-TGCTCTCCCTCTATGCTTCT-3’	
	R: 5’-AGTGCATAGCCCTCGTAGATA-3’	

**Abbreviations:** *ACTG2*, Actin Gamma 2, Smooth Muscle; *ALPI*, Intestinal Alkaline Phosphatase; *CLDN3*, Claudin-3; F: Forward primer; *FABP2*, Fatty Acid Binding Protein 2; R: Reverse primer; *TFF3*, Trefoil Factor Family 3. *Numbers in brackets [37] indicate the reference source for the corresponding primer sequence.

#### 2.7.4. Molecular screening for viral enteric pathogens

To ensure that the observed mucosal alterations and gene expression profiles were specifically associated with *G. duodenalis* and not confounded by viral co-infections, all intestinal samples were screened for common viral enteric pathogens. Total RNA extracted from the paraffin-embedded tissues was subjected to RT-qPCR analysis for rotavirus and coronavirus, using the described primers (Table 3) and protocols.

### 2.8. Statistical analysis

Data obtained from the molecular analyses were evaluated using the SPSS 22.0 statistical software. ANOVA and Games-Howell tests were applied for statistical comparisons. The graphs were created using GraphPad Prism 10.6.1.

## 3. Results

### 3.1. Histopathological findings

**Figure 1.**
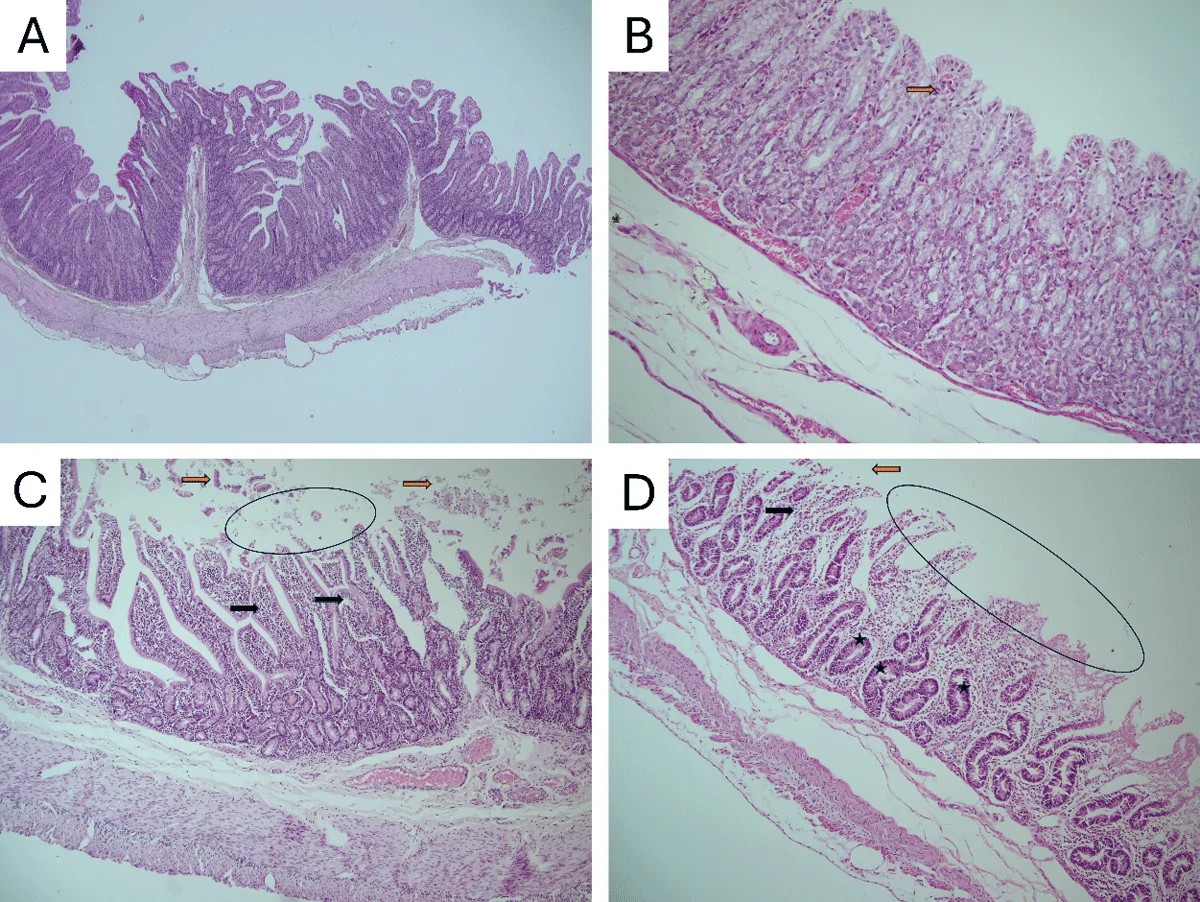
**A:** Control group showing normal villus/crypt ratio and intact epithelial layer integrity. ×4, HE. **B:** Mild villus shortening, mild lymphoplasmacytic cell infiltration (orange arrow), and hyperemia in the lamina propria. ×20, HE. **C:** Moderate villus shortening (ellipse), widespread degeneration and desquamation in the intestinal epithelium (orange arrows), marked lymphoplasmacytic infiltration in the lamina propria (black arrows), and hyperemia. ×10, HE. **D:** Severe villus atrophy (ellipse), extensive necrosis and epithelial loss in the intestinal epithelium (orange arrow), severe lymphoplasmacytic infiltration in the lamina propria (black arrows), and marked crypt hyperplasia (stars). ×10, HE.

**Graph 1.**
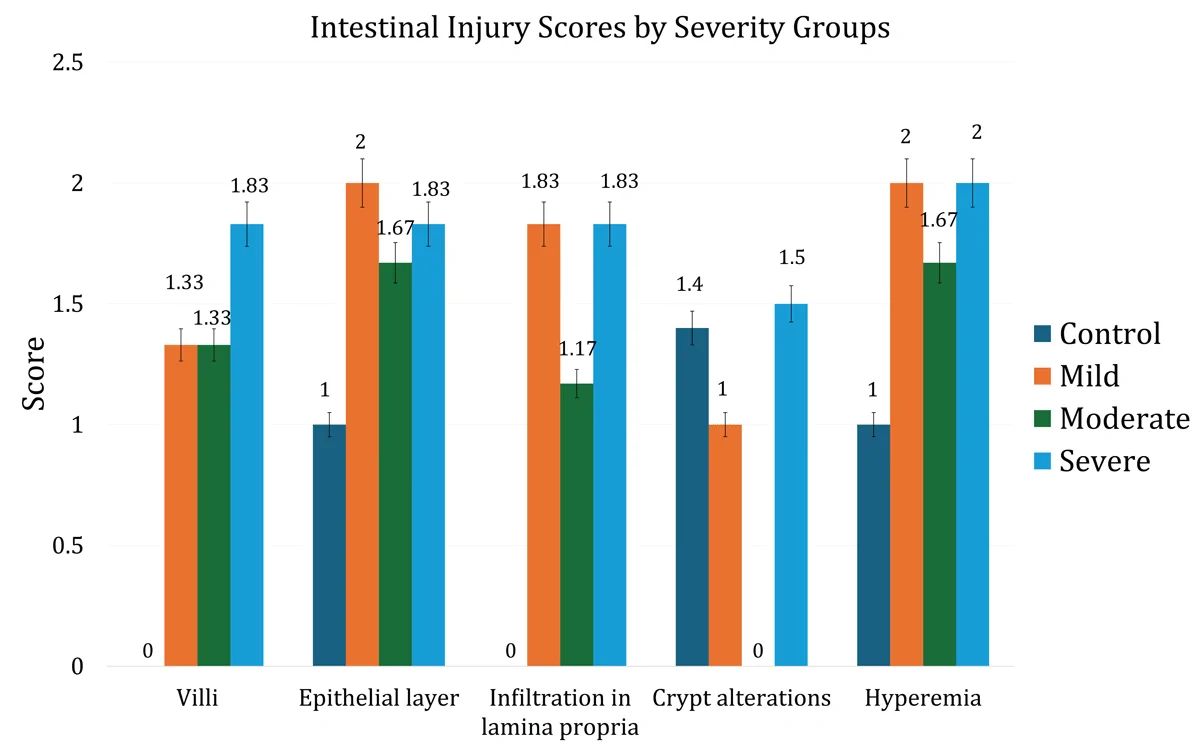
Quantitative assessment of histopathological intestinal injury across different severity groups (mean ± SEM).

The histopathological appearance of the intestines is presented in Figure 1, and the findings obtained from the histopathological scoring are shown in Table 6 and Graph 1.

**Table 3.** Primer sequences used for the detection of rotavirus and coronavirus by PCR.

Rotavirus	F: RTGACAGAGTCGTTAAAGAAATGAGACG	[38]
	R: RTTCCACTCTCCCATTCTTCTAGCG	
Coronavirus	F: RTCATCCCGCTTCACTGATCTCTTG	
	R: RTACTGCCTAGGATACAACACGTCC	

**Table 4.** Reaction mixture preparation procedure.

Component	Volume
Master mix	10 µl
Forward Primer	0.5 µl
Reverse Primer	0.5 µl
dH_2_O	4 µl
Total volume	15 µl

**Table 5.** Real-time PCR reaction conditions.

Step	Temperature	Duration	Cycle number
Pre-incubation	95^o^C	3 min	1
Amplification			
Denaturation	95^o^C	15 sec	40
Annealing	60^o^C	60 sec	
Cooling	40^o^C	30 sec	1

**Table 6.** Histopathological statistics of the intestines*.

Groups	Villi	Epithelial layer	Infiltration in lamina propria	Crypt Alterations	Hyperemia
Control	0.00 ± 0.00^a^	1.00 ± 0.00^a^	0.00 ± 0.00^a^	0.00 ± 0.00^a^	1.00 ± 0,00^a^
Mild	1.33 ± 0.42^b^	2.00 ± 0.37^b^	1.83 ± 0.31^b^	1.40 ± 0.40^b^	2.00 ± 0.52^b^
Moderate	1.33 ± 0.42^b^	1.67 ± 0.49^b^	1.17 ± 0.31^b^	1.00 ± 0.00^b^	1.67 ± 0.49^b^
Severe	1.83 ± 0.40^b^	1.83 ± 0.40^b^	1.83 ± 0.48^b^	1.50 ± 0.43^b^	2.00 ± 0.52^b^

*Data are presented as mean ± SEM (standard error of the mean). ^a, b,^ Different superscript letters within the same column indicate statistically significant differences between groups (*p* < 0.05).

### 3.2. Real-time PCR findings

**Graph 2.**
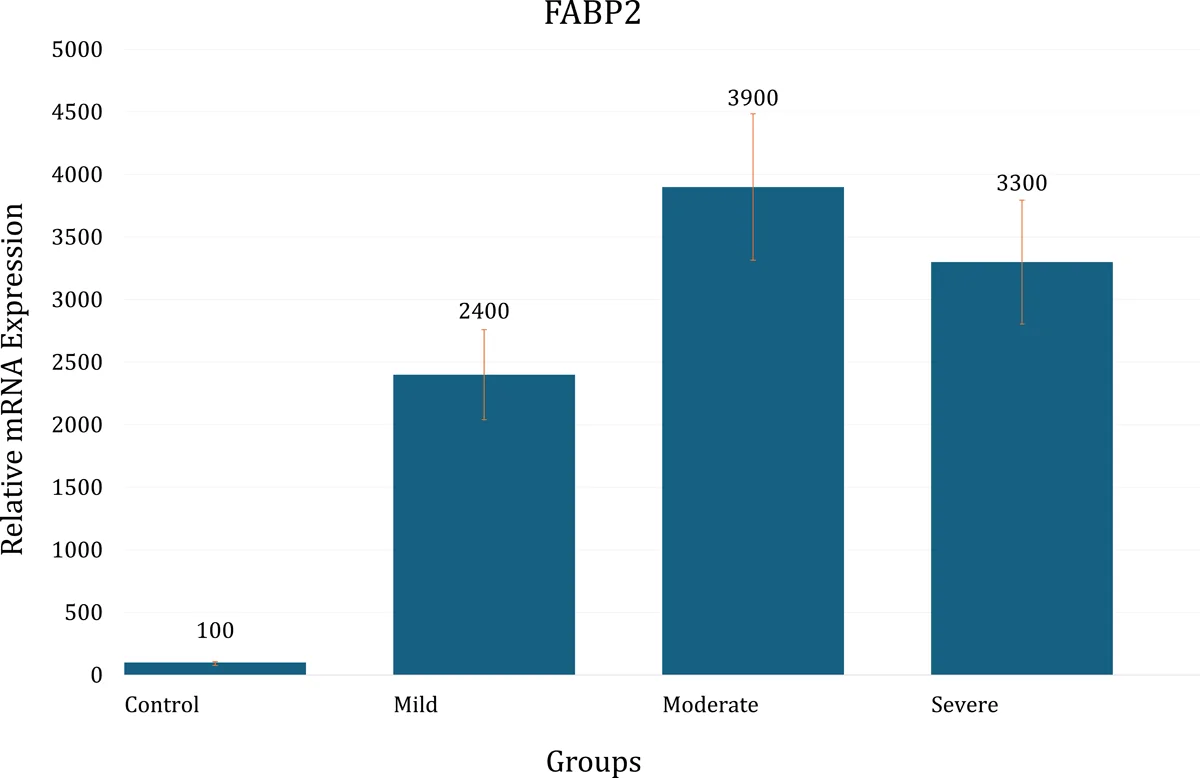
*FABP2* gene expression levels by groups (mean ± SD).

**Graph 3.**
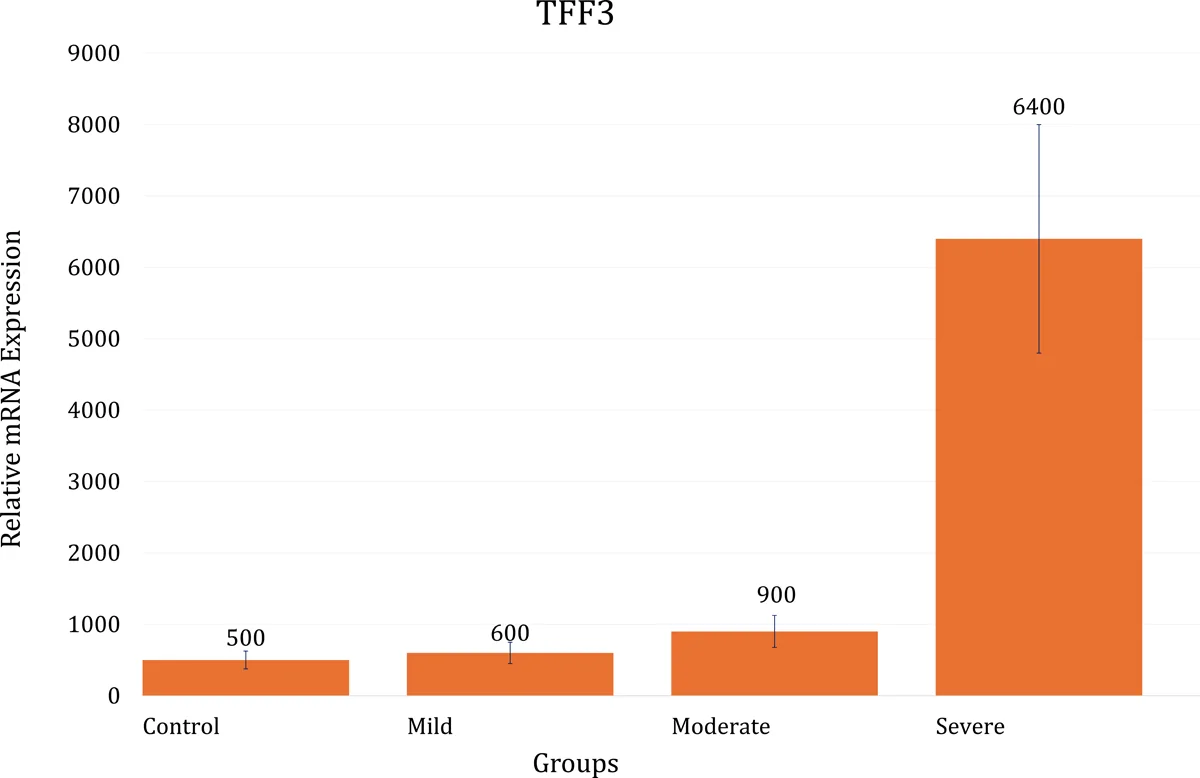
*TFF3* gene expression levels by groups (mean ± SD).

**Graph 4.**
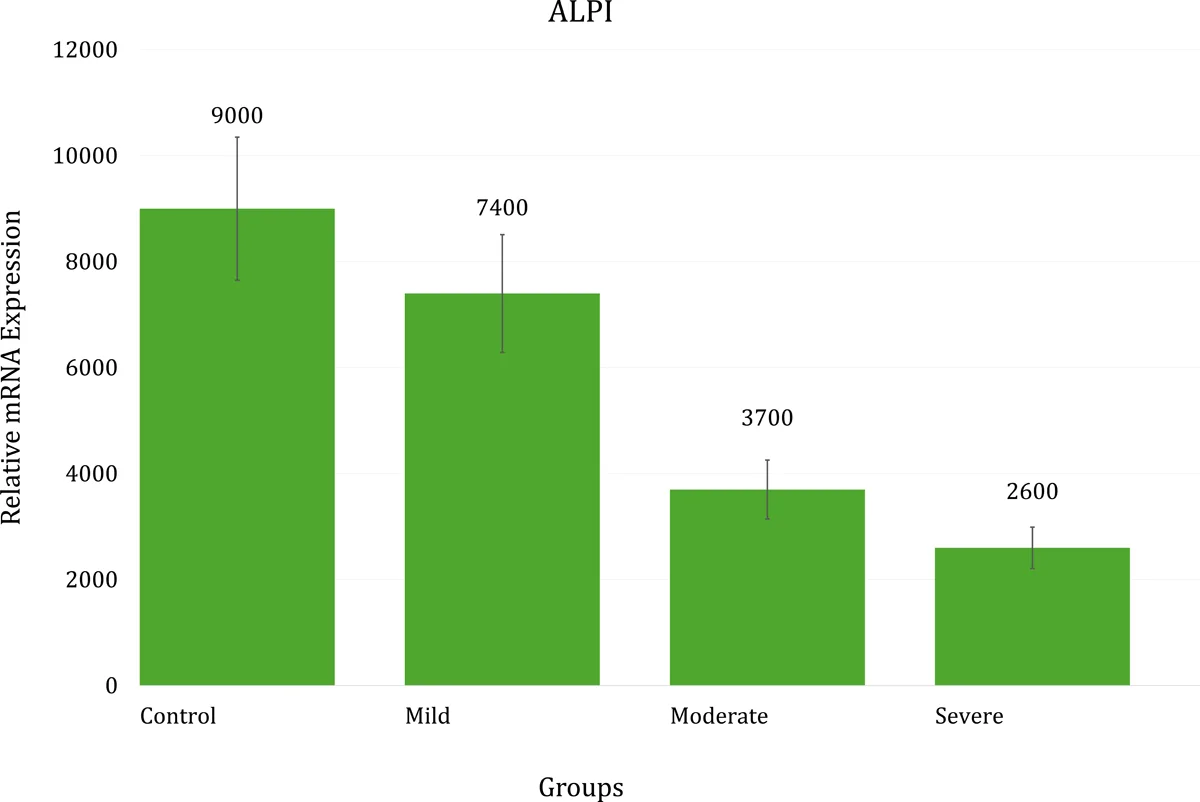
*ALPI* gene expression levels by groups (mean ± SD).

**Graph 5.**
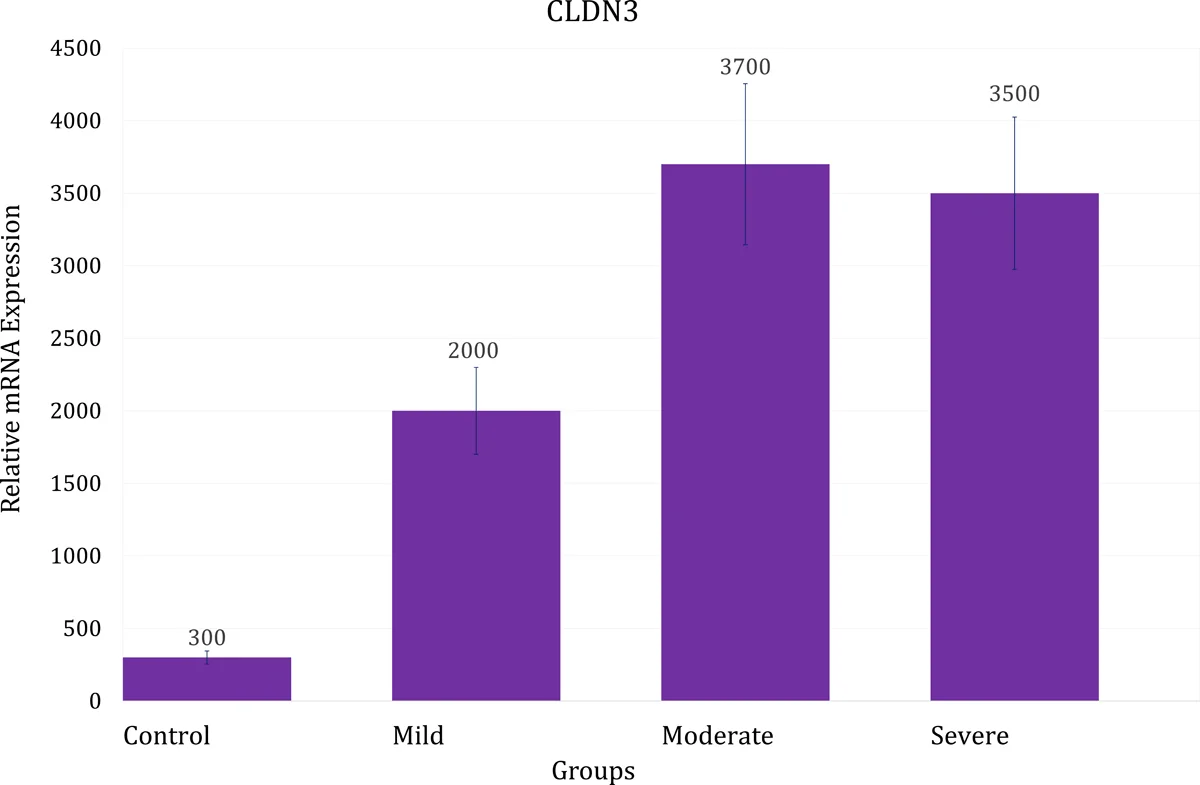
*CLDN3* gene expression levels by groups (mean ± SD).

**Graph 6.**
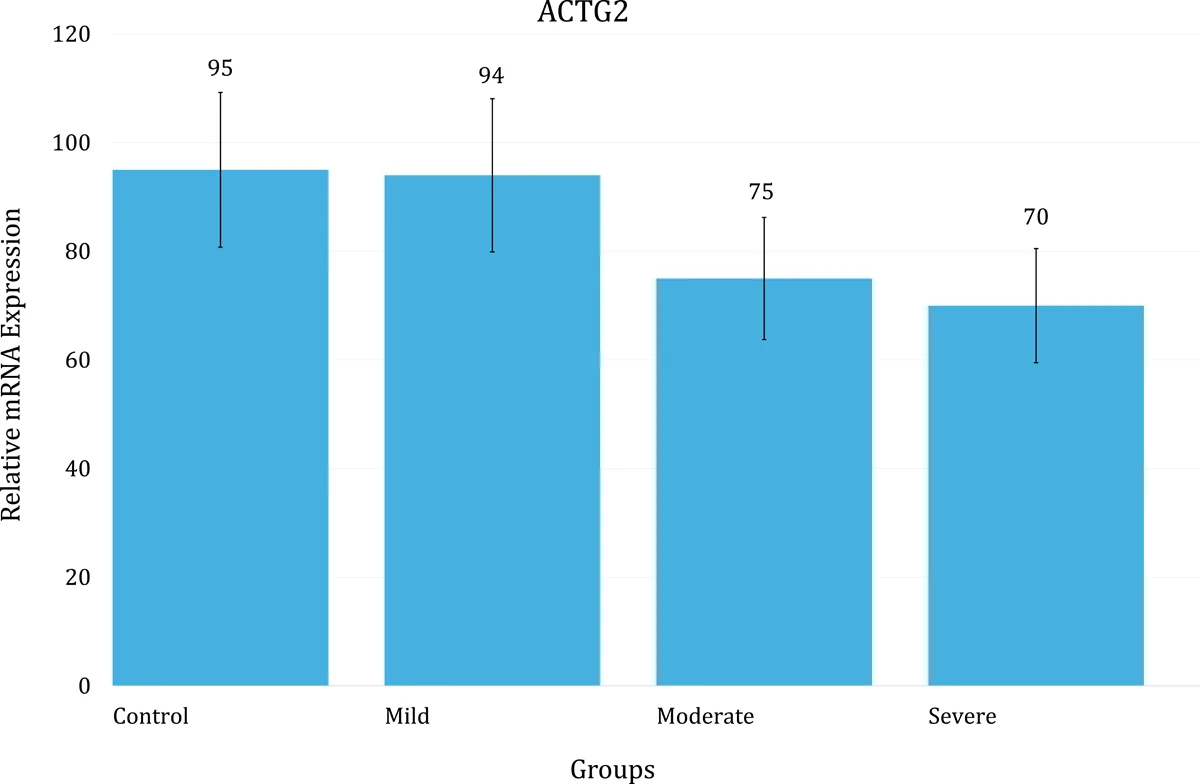
*ACTG2* gene expression levels by groups (mean ± SD).

The statistical analysis of the results obtained from the Real-Time PCR analysis is presented in Graph 2, Graph 3, Graph 4, Graph 5, and Graph 6.

#### 3.2.1. FABP2

According to the statistical evaluation, the results of the one-way ANOVA indicated a significant difference among groups in *FABP2* expression levels (*p* < 0.01). Based on the Games–Howell multiple comparison test, the control group showed significantly lower FABP2 levels compared to the mild (*p* < 0.05), moderate (*p* < 0.01), and severe (*p* < 0.05) groups (Graph 2).

#### 3.2.2. TFF3

A highly significant difference was detected among the groups for the *TFF3* gene (*p* < 0.001). According to the Games–Howell test, the control group exhibited significantly lower expression compared to the moderate (*p* < 0.01) and severe (*p* < 0.05) groups. In addition, the severe group showed markedly higher expression levels than both the control and mild groups (*p* < 0.05) (Graph 3).

#### 3.2.3. ALPI

A significant difference was observed among the groups in *ALPI* expression (*p* < 0.01). In the Games–Howell post-hoc analysis, a decreasing trend approaching statistical significance was detected between the control group and both the moderate (*p* = 0.064) and severe (*p* = 0.051) groups (Graph 4).

#### 3.2.4. CLDN3

According to the ANOVA results, *CLDN3* expression also showed a significant difference among the groups (*p* = 0.001). The Games–Howell test revealed that the control group had significantly lower CLDN3 levels compared to the mild (*p* < 0.01), moderate (*p* < 0.05), and severe (*p* < 0.05) groups. No statistically significant differences were observed among the groups themselves (Graph 5).

#### 3.2.5. ACTG2

In terms of *ACTG2* expression, no significant difference was found among the groups (*p* > 0.5). The Games–Howell comparisons also showed *p*-values > 0.5 for all pairwise analyses, indicating that there were no statistically significant differences between any of the groups (Graph 6).

## 4. Discussion

To the best of our knowledge, this is the first study to integrate histopathological grading with the mRNA expression of specific intestinal biomarkers (*FABP2, TFF3, ALPI, CLDN3*, and *ACTG2*) to elucidate the molecular pathogenesis of natural giardiasis in lambs. As one of the most intricate and evolutionarily sophisticated pathogens of the gastrointestinal tract, *Giardia duodenalis* compromises intestinal barrier function through complex molecular mechanisms rather than mere physical presence. The present study evaluates the interplay between histopathological findings and alterations in molecular signaling pathways in lambs naturally infected with *G. duodenalis*, framed within the “damage-defense-repair” axis.

The observed villous atrophy and crypt hyperplasia in histopathological examinations represent structural manifestations of enterocyte apoptosis induced by the parasite’s attachment to the intestinal epithelium. Such a dramatic decline in the villus-to-crypt ratio signifies more than a mere morphological deformity; it indicates a functional exhaustion or near-total loss of the intestinal absorptive surface area. Furthermore, the dense lymphoplasmacytic infiltration identified within the lamina propria corroborates a persistent inflammatory host response elicited by the parasite’s secreted proteases and structural antigens.

The significant elevation of *FABP2*—a definitive indicator of cytosolic integrity in intestinal epithelial cells—across mild, moderate, and severe groups demonstrates its potential as a potent “early-stage mucosal injury sensor” in giardiasis. Our findings regarding this elevation align with the role of *FABP2* as a sensitive marker of enterocyte injury, acting as a molecular “distress signal” for villous atrophy and epithelial turnover triggered by the mechanical and enzymatic disruption of *Giardia* trophozoites. These results are consistent with the pioneering study by Ok et al. [27], who identified *FABP2* as a reliable biomarker for detecting intestinal epithelial damage in neonatal calves with diarrhea. Intriguingly, the stabilization of this increase observed in our severe group suggests an exhaustion of the healthy enterocyte population capable of synthesizing *FABP2*, likely due to advanced cellular loss in terminal stages. This pattern underscores the capacity of *FABP2* to serve as a high-fidelity biomarker for detecting occult mucosal damage, even in subclinical cases that remain asymptomatic.

The most novel and noteworthy finding of this study is the marked upregulation of *CLDN3* expression across all infected groups (*p* = 0.001). While a vast majority of current literature—primarily based on human and murine models—reports that Giardia induces a “leaky gut” condition by downregulating tight-junction proteins, our data suggest a distinct pathophysiological trajectory in lambs. It appears that the host does not remain passive against the *G. duodenalis* assault; instead, it exhibits a robust compensatory resistance by synthesizing additional *CLDN3* to fortify the intestinal barrier. This “defense paradox” may be interpreted as a species-specific molecular armor strategy developed by ruminants through evolutionary adaptation to giardiasis. Such genetic resilience is likely a critical determinant of the host-parasite interplay, in which the ruminant gastrointestinal tract is programmed to maintain paracellular integrity rather than succumb to trophozoite-induced disruption. This genetically driven barrier fortification may explain why many small ruminants remain subclinical despite significant parasitic burdens, highlighting the importance of host genetic background in the molecular pathogenesis of enteric protozoal infections.

In addition to the structural damage, the host initiates a vigorous restorative response through *TFF3* secretion. We observed that *TFF3* expression levels peaked at the stage of the most severe tissue damage (*p* < 0.001), serving as a robust indicator of the organism’s survival effort under substantial pathological stress. Secreted by goblet cells, *TFF3* functions as a dual-action agent: it stabilizes the protective mucus layer to restrict the parasite’s attachment sites while simultaneously triggering epithelial cell migration to seal the molecular gaps induced by the infection [13, 14]. Our data showing this pronounced *TFF3* upregulation suggests that the lamb’s intestine maintains a resilient capacity for self-renewal despite the parasitic burden. This observation is consistent with the general ruminant enteric defense mechanisms discussed by Alhallaq & Ok [29], who highlighted that trefoil factors play a pivotal role in limiting the spread of luminal pathogens and maintaining mucosal integrity in neonatal lambs. Thus, the vigorous *TFF3* response identified in our study provides concrete evidence of a functional, though stressed, mucosal repair machinery during the severe phase of giardiasis.

Conversely, the marked reduction in *ALPI* expression represents perhaps the most devastating and clinically significant outcome of the infection. Rather than being a mere enzyme, *ALPI* functions as a critical defensive agent that neutralizes bacterial endotoxins (LPS) within the intestinal lumen, modulates inflammation, and orchestrates lipid absorption [34, 35, 36]. The significant downregulation observed in our moderate and severe groups suggests a catastrophic failure in the mucosal detoxification barrier. This deficit provides a clear molecular basis for the diarrhea, steatorrhea, and growth retardation clinically observed in these lambs, leaving the organism vulnerable not only to the parasite itself but also to secondary opportunistic pathogens—a mechanism also highlighted in other protozoal models, such as coccidiosis in calves [28]. In our study, no significant difference was observed in *ACTG2* expression—associated with smooth muscle contraction and structural integrity—among the groups (*p* > 0.05). This finding holds substantial pathophysiological importance, as it demonstrates that the tissue destruction induced by *Giardia* does not extend into the tunica muscularis or deep smooth muscle fibers. Instead, the pathological damage appears strictly confined to the mucosal and submucosal layers. Consequently, we can conclude that the diarrhea and motility alterations observed in giardiasis are likely attributed to osmotic imbalances within the intestinal lumen and the functional impact of inflammatory mediators, rather than direct structural injury to the intestinal smooth muscle.

The molecular dynamics identified in our study strongly suggest that giardiasis in lambs transcends a simple parasitic process, establishing a molecular substrate that facilitates the pathogenesis of enteric viral agents such as rotavirus and coronavirus. Specifically, the declining trend in *ALPI* expression indicates a weakened antiviral and antitoxic defense barrier within the intestinal lumen. Concurrently, fluctuations in *CLDN3* protein levels provide evidence of compromised tight-junction integrity, which potentially paves the way for viral translocation into the submucosa. The elevated *TFF3* expression observed in the severe group likely represents a final compensatory repair effort to mitigate the profound epithelial necrosis resulting from a viral burden superimposed on the parasitic load. Taken together, the extreme genetic shifts and severe clinical manifestations in certain lambs should be viewed as a tangible consequence of a parasite-virus co-infection, shaped by the molecular instability *Giardia* induces within the intestinal mucosa. Consequently, the management of giardiasis emerges as a strategic priority for maintaining the overall viral resilience of the flock. Our molecular findings align with previous research in other ruminant models. For example, the significant fluctuations in *FABP2* levels observed in our infected lambs are consistent with the epithelial damage patterns reported by Ok et al. [27] and Durgut & Ok [28] in diarrheic calves. Similarly, the activation of mucosal repair and barrier markers in our study supports the diagnostic framework established by Gao et al. [36] in neonatal lambs. By building upon these existing studies, our results further confirm that intestine-related biomarkers are potent tools for tracking the progression of protozoal-induced enteropathy.

In summary, this study demonstrates that the pathogenesis of giardiasis transcends localized parasitic damage, representing a comprehensive ‘total defense struggle’ by the host at the molecular level. Within this framework, elevations in *FABP2* and *CLDN3* function as early warning signals and barrier fortifications, respectively; the increase in *TFF3* signifies emergency mucosal repair, while the decline in *ALPI* represents a critical metabolic breach. These data establish a robust foundation for the development of “barrier-oriented therapeutic” strategies. Such future interventions should not only focus on antiparasitic agents but also incorporate probiotics and specific enzyme supplements designed to bolster protective molecules like *ALPI* and *TFF3*.

## 5. Conclusions

To conclude, our findings clearly illustrate that natural *Giardia duodenalis* infection in lambs triggers a sophisticated intestinal response where mucosal destruction and molecular regeneration occur simultaneously. We observed that the severity of histopathological lesions—specifically villus atrophy—parallels the fluctuations in *FABP2, TFF3*, and *ALPI* expression, suggesting these markers accurately reflect the degree of enterocyte damage. Notably, the unexpected rise in *CLDN3* levels points toward a robust compensatory effort by the host to counteract tight junction leakage and maintain the gut barrier during active infection. By identifying *FABP2* and *TFF3* as sensitive molecular indicators, this study offers a new diagnostic perspective for monitoring giardiasis-induced mucosal healing in sheep. Moving forward, investigating the link between these expression profiles and actual weight gain losses will be vital for optimizing clinical management in infected herds.

## Data Availability

The data presented in this study are available from the corresponding author upon reasonable request.

## List of abbreviations

ACTG2, actin gamma 2, smooth muscle; ALP, alkaline phosphatase; ALPI, intestinal alkaline phosphatase; CLDN3, claudin-3; DNA, deoxyribonucleic acid; ELISA, enzyme-linked immunosorbent assay; FABP2, fatty acid binding protein 2; HE, hematoxylin and eosin; LPS, lipopolysaccharides; mRNA, messenger ribonucleic acid; PCR, polymerase chain reaction; qPCR, quantitative polymerase chain reaction; rRNA, ribosomal ribonucleic acid; TFF1, trefoil factor 1; TFF2, trefoil factor 2; TFF3, trefoil factor family 3.
